# Use of Computer Vision with Conventional Video Recordings of Gait in Older Adults: A Scoping Review

**DOI:** 10.3390/healthcare14162487

**Published:** 2026-08-11

**Authors:** Chitra Banarjee, Anthony Bilic, Yong Jae Kwon, Sofia Rojas Aristizabal, Janet Lopez, Rui Xie, Chen Chen, Ladda Thiamwong

**Affiliations:** 1 College of Medicine, University of Central Florida, Orlando, FL 32827, USA; 2 College of Nursing, University of Central Florida, Orlando, FL 32827, USA; sofia.rojasaristizabal@ucf.edu (S.R.A.); janet.lopez@ucf.edu (J.L.); rui.xie@ucf.edu (R.X.); ladda.thiamwong@ucf.edu (L.T.); 3 Institute of Artificial Intelligence, University of Central Florida, Orlando, FL 32816, USA; anthony.bilic@ucf.edu (A.B.); yo564250@ucf.edu (Y.J.K.); chen.chen@crcv.ucf.edu (C.C.); 4 Disability, Aging, and Technology Cluster, University of Central Florida, Orlando, FL 32827, USA

**Keywords:** mobility, geriatrics, pose estimation, screening, walking

## Abstract

**Highlights:**

**What are the main findings?**

- This paper identified 46 studies that use computer vision (CV) with conventional video recordings to analyze gait in older adults, predominantly focusing on screening, classification, and characterization of older adult gait using short walking tasks.
- Validation approaches were inconsistent across studies: most compared CV outputs to clinical labels or diagnoses, while fewer validated against gold standards.

**What are the implications of the main findings?**

- CV-based gait analysis using conventional cameras has demonstrated technical feasibility and preliminary performance for screening and classification in older adults; however, heterogeneous methods, insufficient external validation, and the near absence of longitudinal or home-based studies prevent conclusions about readiness for routine clinical use.
- Standardized reporting protocols and interdisciplinary validation studies in community-dwelling older adults without specific pathology are needed before video-based gait tools can reliably inform clinical decision-making.

**Abstract:**

**Background/Objectives**: This scoping review evaluates the use of computer vision (CV) for analyzing conventional video recordings of gait in older adults. In the rapidly evolving world of CV, methods that use conventional video recordings offer a practical and affordable solution to quantifying gait impairments, especially in resource-limited areas. In this review, we evaluate the aims, methods, variables, and validation of the literature utilizing videos of gait in older adults. **Methods**: Inclusion criteria were limited to journal articles and conference papers published between 2020 and 2025. Three reviewers extracted data from selected articles. **Results**: The search identified 46 studies that demonstrate a wide variety of clinical conditions, methodological approaches, and analyzed variables. Three main aims emerged, with 15 screening studies for clinical conditions, 14 studies classifying severity or mobility levels, 16 characterization studies of healthy and pathological gait, and one person-identification study. Methodological approaches ranged across 35 studies utilizing commercially available cameras, six studies utilizing conventional video from specialized cameras, two using surveillance-style cameras, and four that did not specify. Methods of validation were inconsistent, comparing either to lab-based biomechanical gold standards or broad clinical labels, with limited interdisciplinary validation through both methods. **Conclusions**: This scoping review demonstrates methodological growth and diversification in CV-based gait analysis using conventional video in older adults. The available evidence primarily supports technical feasibility and preliminary screening or classification performance rather than established clinical validity. Standardized methods and external, longitudinal, and real-world validation are needed before these approaches can reliably support routine clinical decision-making.

## 1. Introduction

Computer vision (CV) is a powerful and rapidly evolving tool with a wide range of healthcare applications [1]. It has been used to evaluate medical images, ranging from magnetic resonance imaging and computed tomography scans to images of retinas and skin lesions [2,3,4,5]. In addition to medical images, computer vision can be harnessed to extract useful information from conventional video recordings, including the dynamics of human movement [6,7]. Several studies have explored the validation and development of CV applications for gait [8,9,10]. Here, we focus on the use of CV on conventional video recordings for gait analysis in older adults to understand the current applications and potential for implementation in healthcare settings.

Movement is central to human interaction and well-being across the lifespan [11,12,13]. Both of these are especially critical for older adults to maintain their social connections, activities of daily living (ADLs), and cognitive functioning [14,15,16]. Evaluations and interventions focused on gait have the potential to decrease fall risk, improve neuromuscular disorders, and combat loneliness [17,18,19]. Both lab-based and clinical assessments of gait have limitations. Laboratory research evaluates human movement using wearable sensors, infrared marker-based systems, and specialized cameras [7,20,21], which widely differ from the typically observational evaluation of gait in clinical settings [22,23,24]. Clinical assessment of gait typically utilizes a trained observer with a stopwatch or a target distance and is evaluated with subjective ratings and non-specific cutoffs [23,25]. While these approaches are widely used, they can be subjective and limited in the level of detail they capture. CV offers the ability to transform conventional video recordings into objective, quantitative assessments of human movement to improve gait evaluation.

Early scientific analysis of human movement through vision systems can be dated up to the 1980s, when Marr’s computational theory of vision formalized hierarchical representations for interpreting human body shape and motion [26]. With the rise in digital computing and CV, movement analysis evolved into articulated model-based tracking systems that represented the body as a kinematic chain, eventually influencing gait biometrics such as the Gait Energy Image [27]. The deep learning era marked a major turning point: convolutional models such as DeepPose [28,29] and OpenPose [30] enabled robust 2D pose estimation from RGB images in unconstrained environments. These methods reframed movement analysis as large-scale supervised learning rather than handcrafted geometric fitting.

Subsequent advances extended estimation from 2D keypoints to full 3D human reconstruction through parametric body models such as skinned multi-person linear (SMPL) models [31] and end-to-end monocular human mesh recovery [32]. Temporal modeling approaches, including spatiotemporal graph convolutional networks [33] and VideoPose3D [34], further improved motion consistency and dynamic inference. Parallel to these algorithmic developments, systematic reviews of markerless motion capture in rehabilitation [35] and clinical validation studies in stroke [36] and single-camera gait analysis [37] demonstrated growing medical applicability. Together, these works trace the progression from perceptual motion studies to data-driven, temporally aware models capable of supporting clinically meaningful movement assessment using monocular RGB video. By translating visual observations into structured metrics, CV provides a more consistent and reproducible foundation for clinical assessment and supports data-driven decision-making in rehabilitation and geriatric care.

Beyond methodological advances, CV offers practical advantages that make it particularly well-suited for gait assessment in older adults. Unlike laboratory-based motion capture systems or instrumented walkways, CV approaches can operate on conventional video recordings acquired with widely available cameras, reducing cost, technical burden, and infrastructure requirements [38,39]. This enables gait assessment in clinical settings with limited resources as well as in home and community environments, where falls most often occur [40]. In addition, video-based data can be collected repeatedly and reanalyzed over time as computational methods evolve, supporting scalable and longitudinal monitoring of mobility, disease progression, and treatment response [41]. These characteristics position CV as a promising avenue for expanding access to objective, quantitative gait assessment in geriatric care.

Despite this potential, the existing literature applying CV to gait analysis in older adults remains fragmented and methodologically heterogeneous. Studies vary widely in their aims, CV techniques, extracted gait variables, validation strategies, and reporting practices, making it difficult to synthesize evidence or assess readiness for clinical implementation. Prior reviews have largely focused on novel pose estimation methods, technical validation, or general movement analysis across all ages. However, several gaps remain in the understanding of these tools at the intersection of digital health and geriatrics. Specifically, it is unclear how these methods and tools are being utilized for the evaluation of aging and age-related diseases. Given the rapidly increasing older adult population and the growing adoption of digital health tools, this study aims to fill this gap by focusing on three concepts: (1) older adults, (2) assessment of gait, and (3) conventional video recordings.

This study is guided by an overarching research question: **How has CV been utilized to analyze gait using conventional video recordings of gait in older adults?** The scoping review framework is utilized to capture the breadth of research in this evolving field and identify research priorities for translating this work to clinical practice. This approach is particularly well-suited to emerging fields that have not yet been ‘extensively reviewed or are characterized by complex and heterogeneous evidence’ [42]. Therefore, the review will systematically map how CV has been applied in the geriatric context, identify methodological trends and gaps, and clarify the current state of evidence.

## 2. Materials and Methods

**Figure 1:**
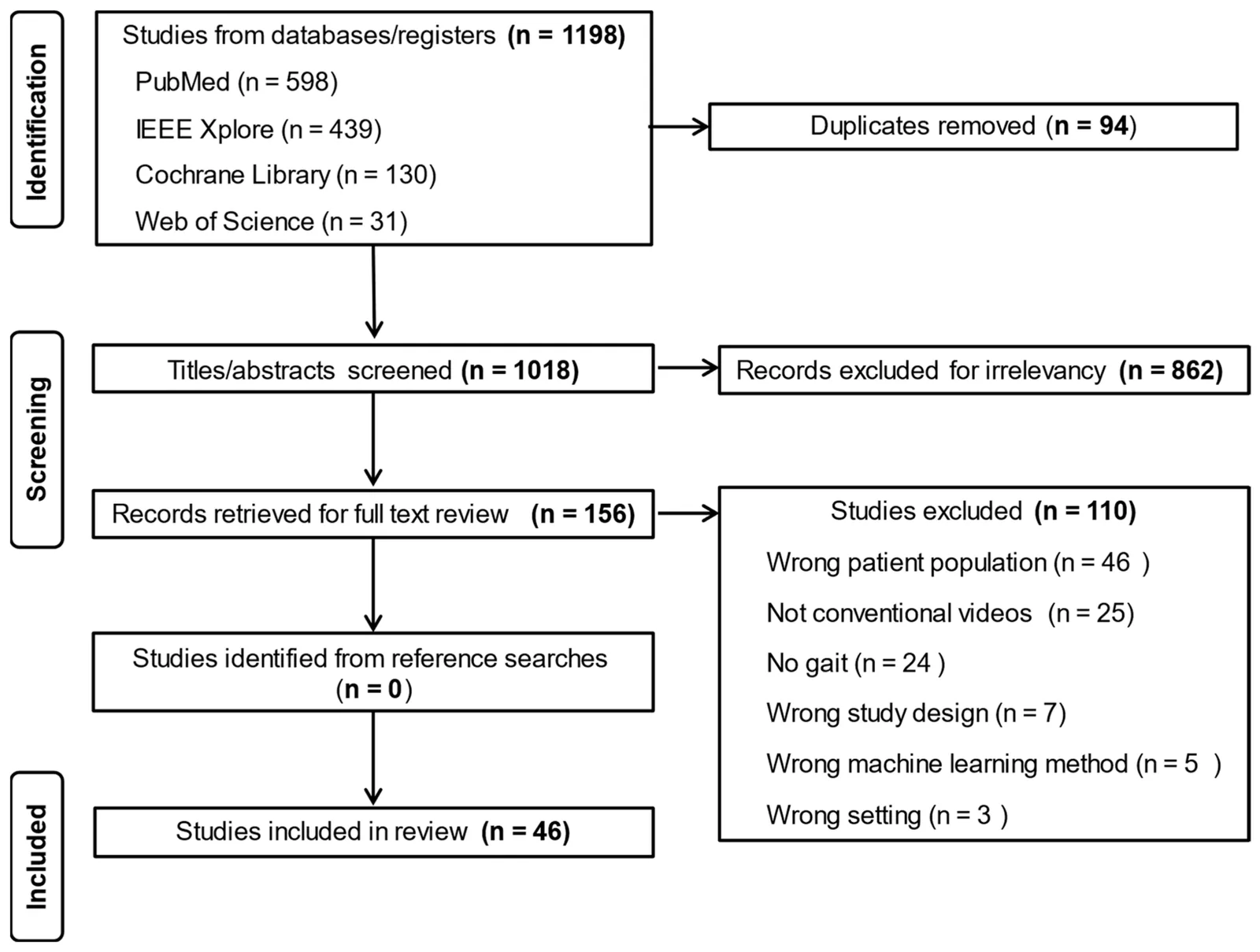
PRISMA-ScR diagram illustrating the scoping review study selection process.

This scoping review was conducted in accordance with Arksey and O’Malley’s methodological framework for scoping reviews [43], which consists of five iterative stages: (1) identifying the research question, (2) identifying relevant studies, (3) study selection, (4) charting the data, and (5) collating, summarizing, and reporting the results. An overview of the review process is presented in Figure 1. Given the rapidly evolving body of literature, a scoping review methodology was selected to map a broad range of methods and applications from CV studies [44]. This review followed the PRISMA extension for Scoping Reviews (PRISMA-ScR) reporting guidelines. There are no human participants, as the review uses data from previously published articles. The protocol for this review was neither registered nor published; however, this study has been retrospectively registered at Open Science Framework (https://osf.io/fjn5g, accessed on 10 August 2026).

### 2.1. Stage 1: Identifying the Research Questions

The primary objective of this scoping review was to examine how CV techniques have been applied to gait analysis using conventional video recordings in older adults. The overarching research question was: How has CV been utilized to analyze gait using conventional video recordings of gait in older adults? To address this question, the following sub-questions guided the review:

1. What are the general aims of CV-based studies that assess gait in older adults?
2. What CV methods, algorithms, or pose estimation techniques are used to extract gait information?
3. What gait-related variables are extracted from video recordings?
4. How is the effectiveness or performance of these methods evaluated or validated?

### 2.2. Stage 2: Identifying Relevant Studies

We consulted a research librarian to guide our database selection and search strategy. A comprehensive literature search was then conducted across four electronic databases (PubMed, IEEE Xplore, Cochrane Library, and Web of Science) to identify relevant studies. In addition, reference lists of included articles were screened to capture any additional eligible studies.

The search was limited to studies published between January 2020 and 13 May 2025. This time frame was selected to reflect the rapid expansion in the availability and use of video-recorded clinical assessments, particularly following the onset of the COVID-19 pandemic, which accelerated the adoption of remote and video-based evaluation methods in clinical and research settings [45,46].

Search terms included variations of “CV”, “gait”, “pose estimation”, “fall risk”, and “older adults”. Search strategies were adapted for each database and information source. The full search strings for each database are included in Supplementary Materials.

### 2.3. Stage 3: Study Selection

#### 2.3.1. Inclusion and Exclusion Criteria

Studies were included if they met the following criteria: (1) included older adults (typically ≥60 years) as the study population; (2) used RGB video-based recording systems, including single-camera (monocular) and multi-camera setups, provided that gait was assessed from standard video data rather than marker-based laboratory motion capture using specialized infrared systems; (3) focused on gait or walking-related tasks relevant to mobility or fall risk assessment; and (4) employed computer-vision or video-based analytical methods (e.g., pose estimation, tracking, or reconstruction algorithms) to extract gait or movement-related information. Systems using multiple synchronized RGB cameras (e.g., for 3D reconstruction such as Theia3D or KinaTrax) were included, as they rely on video-based inputs rather than wearable sensors or marker-based motion capture.

Studies were included if the sample consisted primarily of older adults, operationalized as a mean or median age ≥ 60 years where reported, or if the study explicitly framed its aims in the context of aging, age-related decline, or geriatric populations. Given that several clinical conditions targeted in the literature, including Parkinson’s disease, sarcopenia, and knee osteoarthritis, disproportionately affect older adults, studies focusing on these conditions were included even where full demographic detail was not reported, provided the clinical framing was explicitly geriatric. Studies that enrolled mixed-age samples without stratifying or discussing findings by age were excluded.

Studies were excluded if they (1) did not include a gait or walking component (e.g., studies focused solely on sit-to-stand or stand-to-sit tasks without gait), (2) included only younger adults or mixed age groups without explicit evaluation of older adult gait, and (3) relied exclusively on wearable sensors or non-video-based assessment methods.

Studies were included regardless of design, encompassing both quantitative and qualitative research. Consistent with scoping review methodological standards, we did not evaluate the methodological quality or rigor of the included articles [47]. Only peer-reviewed full-text articles, conference papers, or conference abstracts were included, while white papers, legal documents, protocols, conceptual papers, and editorials were excluded.

#### 2.3.2. Screening Process

Study selection was conducted using Covidence systematic review software (https://www.covidence.org/, accessed on 10 August 2026, Veritas Health Innovation, Melbourne, Australia). Given the volume of records retrieved (n = 1068), title and abstract screening was performed by one trained reviewer to ensure timely completion of the review within available resources. Prior to screening, the review team engaged in initial calibration discussions to align on eligibility criteria and clarify inclusion and exclusion decisions. Ongoing weekly meetings were also held throughout the screening process to address uncertainties and ensure consistent application of the criteria. To mitigate the risk of exclusion errors, the screening criteria were applied conservatively, with ambiguous records advanced to full-text review rather than excluded at this stage. Full-text screening was subsequently conducted independently by two reviewers, with discrepancies resolved through consensus, providing a quality check on the initial screening decisions.

### 2.4. Stage 4: Charting the Data

Data were charted using a structured extraction framework developed iteratively throughout the review process. Through collaboration with all team members, a data extraction template was developed using Covidence and is presented in Supplementary Materials. Team members (CB, AB, YJK) extracted the data, and two team members verified the extraction (CB, SRA). The extracted data were exported to an Excel spreadsheet and examined for completeness, updating missing items as appropriate. Extracted information included study aims, participant characteristics, video acquisition setup, CV or pose estimation methods, extracted gait variables, and evaluation or validation strategies.

### 2.5. Stage 5: Collate, Summarize, and Report the Results

Extracted data were synthesized descriptively and organized according to the scoping review questions. Results are summarized below to characterize trends in study aims, methodological approaches, extracted gait variables, and evaluation strategies, as well as to identify gaps and inconsistencies in the existing literature.

## 3. Results

A total of 46 studies met inclusion criteria, representing a diverse and rapidly evolving body of work applying computer-vision-based methods to human gait analysis across clinical and non-clinical populations. Studies are summarized below in tabular format, and a full table of each study is provided in the Supplementary Materials. Thematic synthesis revealed four major domains aligned with the guiding research questions: (1) aims, (2) methodological approaches, (3) variables extracted, and (4) evaluation and validation strategies.

The majority of excluded studies were due to the wrong patient population (n = 46); these included studies that explored gait analysis in adult or pediatric populations rather than older adults, or studies that included only a small proportion of older adults. Other major groups of excluded studies included several that utilized non-RGB monocular camera systems (n = 25), including the Kinect sensor, which uses RGB-D, adding a depth camera and protocol studies (n = 24) that describe an algorithm without application in a sample of older adults.

### 3.1. Study Characteristics

Age. Across the studies, reporting of age varied. Although gait studies with an emphasis on aging were selected, some of the study samples included younger adults, resulting in a mean age of 66.8 (median 67.1, IQR: 6.8) years. However, seven studies reported a range of ages, rather than a mean, and six studies did not report age.

Sample Size. The sample size also ranged widely from 4 to 462 participants. Across all studies, mean sample size was 82.4 (median: 37.0; IQR: 77.5). Smaller sample sizes were more common in methodological studies, whereas larger samples were typically observed in evaluation studies utilizing existing open-source datasets.

Setting. Of the 46 studies, 19 were conducted in a controlled lab, 15 were conducted in clinical settings, such as a hospital visit or therapy session, 4 in home or residential care facilities, 1 in a gymnasium, 1 in several settings, and 6 did not specify the setting.

Geographic Location. Overall, the dataset shows a geographic concentration in East Asia (China, Japan, Korea, and Taiwan) and Western countries (Canada, United States, and Sweden), with less representation from South Asia, the Middle East, and continental Europe. China was the most represented country, contributing nine studies. Japan contributed seven studies, making it the second most frequent study location. Canada accounted for six studies, representing the largest contribution from North America. The United States, Korea, and Sweden each contributed four studies. Taiwan was represented by two studies. Malaysia contributed two studies. Single studies (n = 1 each) were conducted in Sri Lanka, France, Italy, Iran, Greece, India, Egypt, and Germany.

Publication Type. The review included 30 journal articles and 16 conference papers. Subgroup analyses were performed to summarize study characteristics of the journal articles and conference papers separately, given the uneven evidence base (Supplementary Materials).

### 3.2. Research Aims and Scope

**Figure 2:**
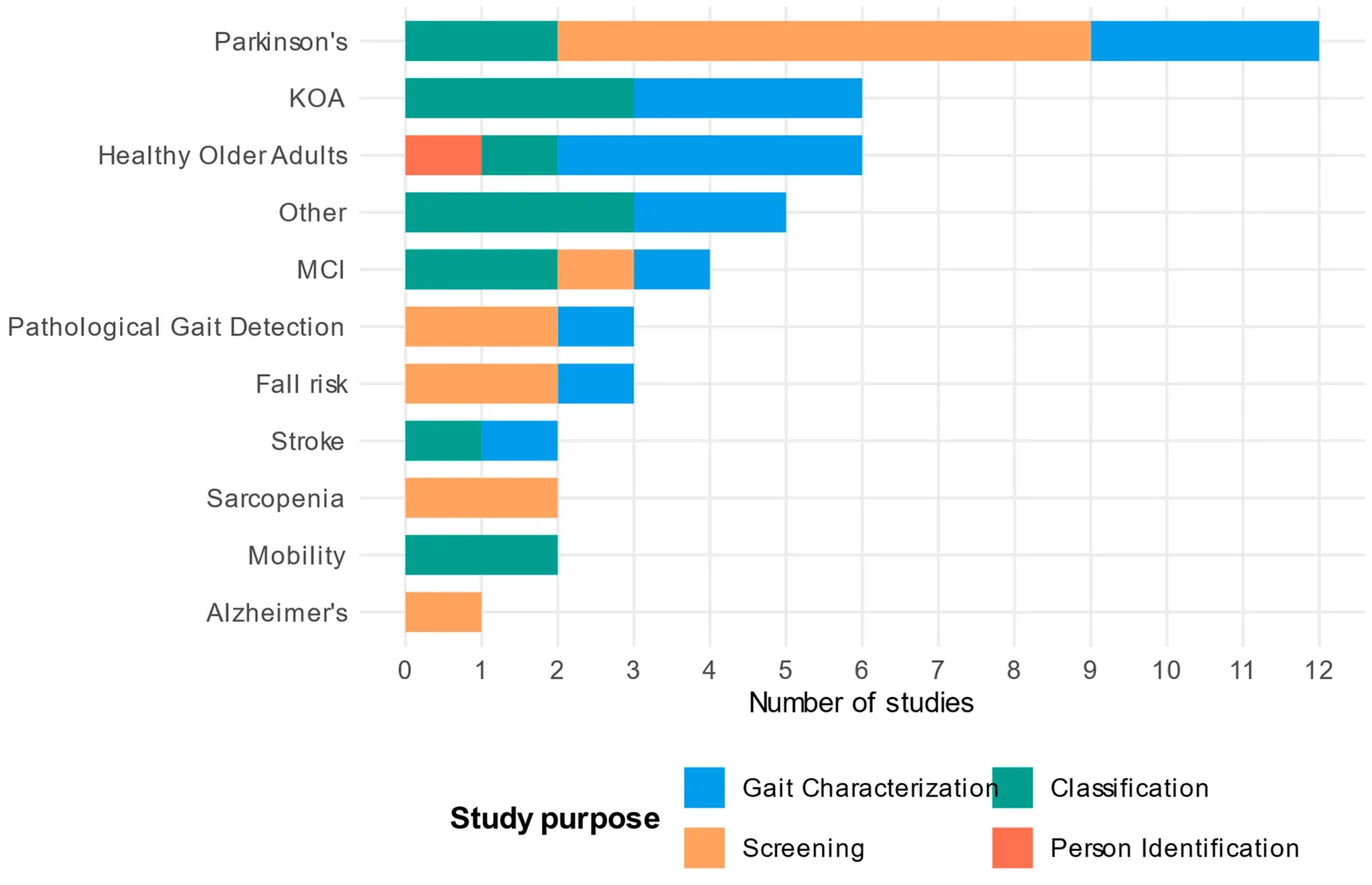
Study purpose by clinical condition. Each bar segment represents one study purpose category.

The included studies spanned four primary domains of application: screening, classification, gait characterization, and single-person identification across several different clinical conditions (Figure 2).

*Screening* made up a total of 15 studies targeting early detection of Parkinson’s disease [48,49,50,51,52,53,54], pathological gait detection [55,56], sarcopenia [57,58], fall risk [59,60], and cognitive impairment (including Alzheimer’s) [61,62]. These studies typically used short walking tasks or timed up-and-go (TUG) videos to differentiate between normal and pathological gait patterns (Table 1).

*Classification* studies extended beyond binary detection to include severity grading (e.g., UPDRS-based severity [63,64,65,66], SARA-based severity in cerebellar ataxia [67], knee osteoarthritis severity and recovery [68,69,70], mobility class categorization [71,72,73,74], and differentiation between neurological disorders such as Parkinson’s disease versus spinocerebellar degeneration [75,76], making up 14 studies.

**Figure 3:**
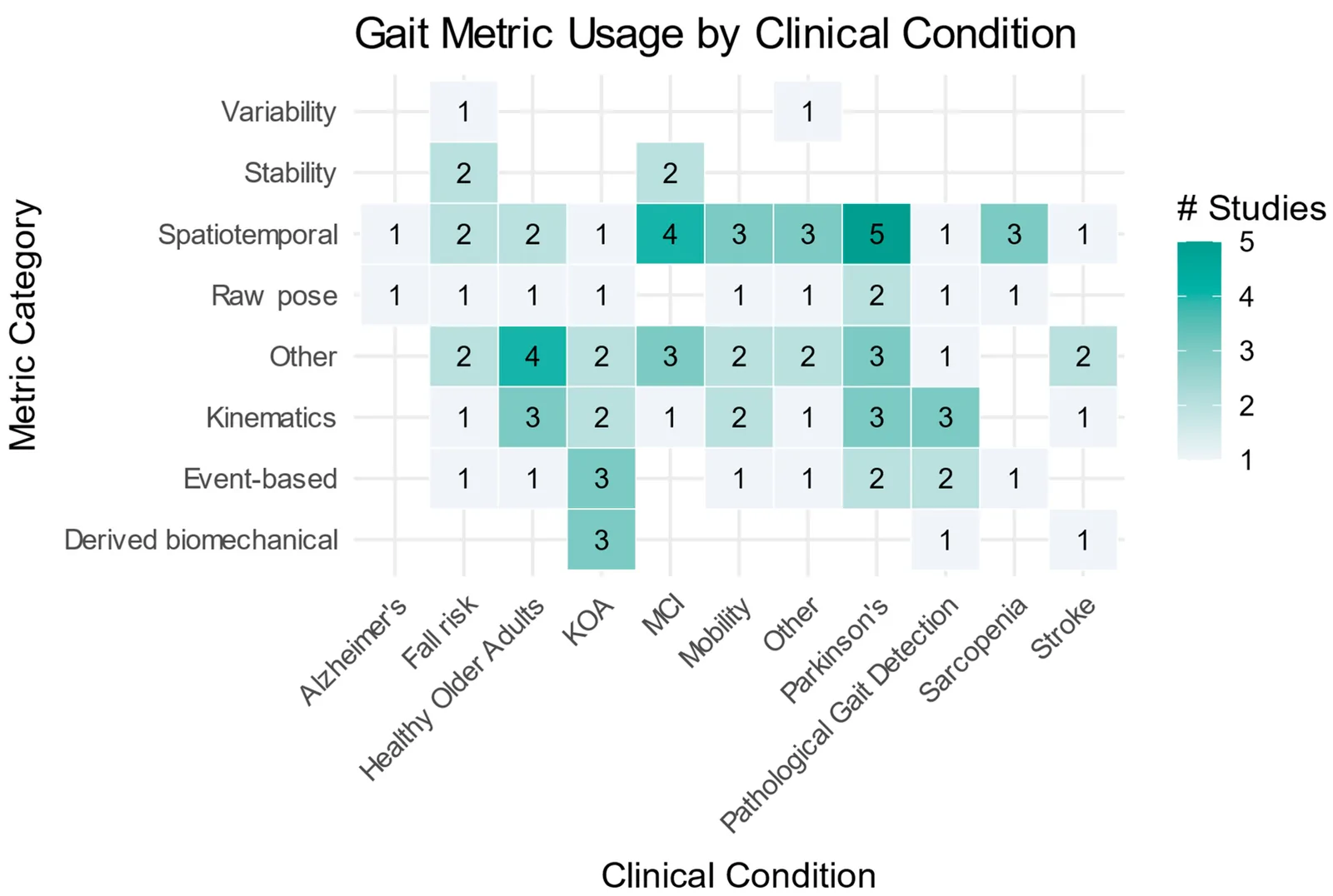
Distribution of gait metric categories across clinical populations.

*Gait characterization* made up 16 studies that quantified spatiotemporal or kinematic features to describe gait signatures without producing diagnostic labels (Figure 3). Gait metrics were summarized into spatiotemporal metrics (step/stride length and width, cadence, speed, stance, and swing times), kinematic metrics (joint-level movement, ranges of motion), event-based metrics (gait events or phase markers), stability metrics (symmetry or margin of stability), variability metrics, derived biomechanical metrics (features computed from models), and raw pose (no feature extraction). Study-specific or non-standard measures not fitting the above categories, including clinical scores (e.g., TUG time, UPDRS) and task-level or composite features, were considered as other metrics.

Finally, one study uniquely addressed single-person identification, using gait signatures extracted from 2D pose estimation to match individuals across videos [77].

### 3.3. Methodological Approaches

#### 3.3.1. Video Recordings

Most studies relied on single-camera RGB video, with 15 studies using smartphones or laptop webcams and 20 using consumer-grade cameras, reflecting an emphasis on accessibility and real-world feasibility. Camera type, number of views, distance, viewing angle, frame rate, and resolution are presented in Supplementary Materials. A smaller subset of six studies employed conventional video recordings from specialized or multi-camera systems, such as KinaTrax and Theia3D, to enhance 3D reconstruction accuracy [55,72,73,78,79,80]. One of the studies that used the Kinect camera also utilized a smartphone [65]. Two studies also used hallway or opportunistic clinical recordings, such as surveillance cameras [59,71], demonstrating interest in deploying these tools in uncontrolled environments. Four studies did not specify their recording device.

#### 3.3.2. Pose Estimation

**Figure 4:**
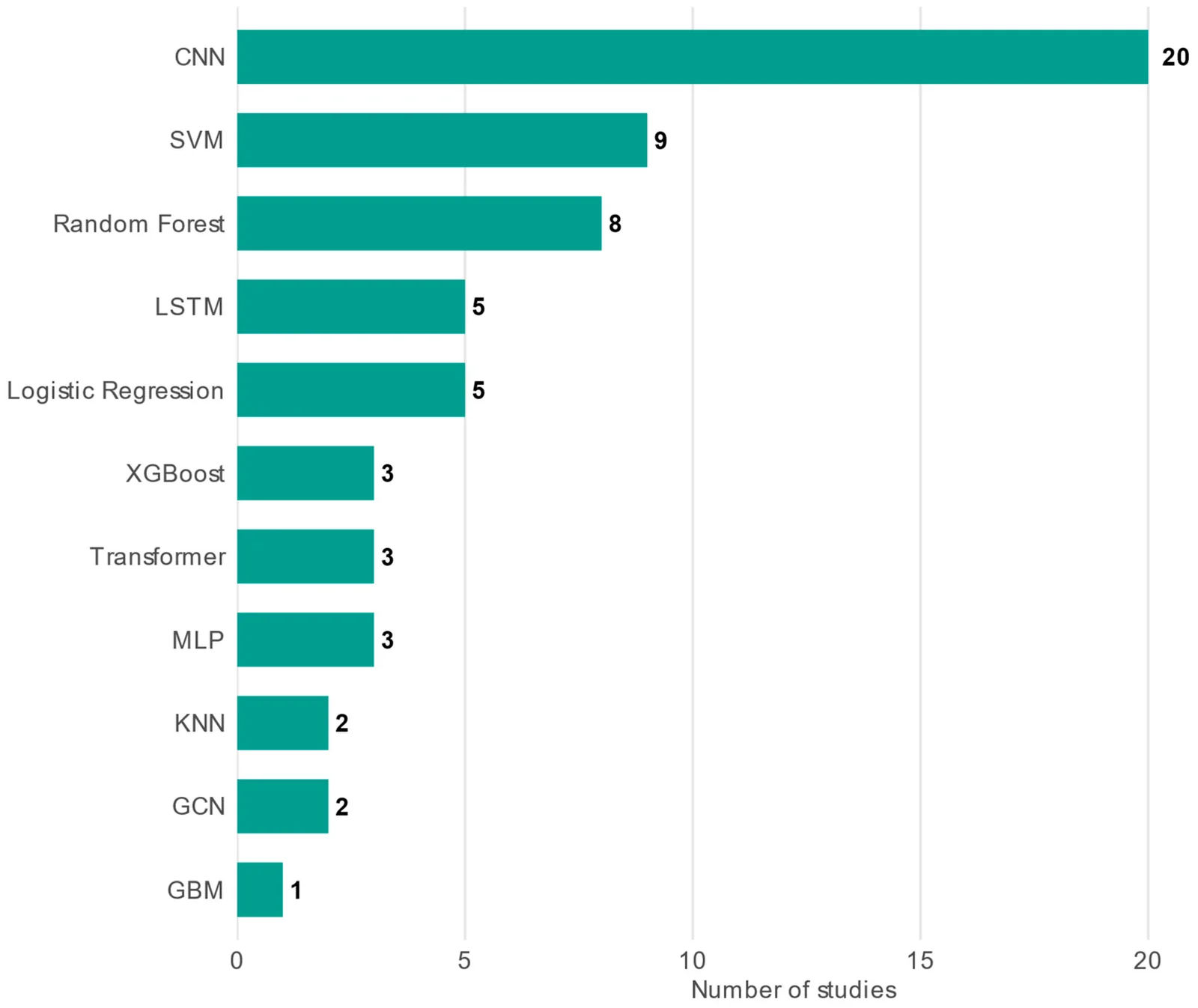
Machine Learning Models. Some studies used multiple types of models.

Across studies, methodological approaches were dominated by 2D pose-estimation frameworks, with OpenPose being the most frequently used tool, followed by MediaPipe/BlazePose, AlphaPose, HRNet, and several commercial systems such as Theia3D, OpenCap, and KinaTrax. These pose-estimation pipelines were commonly paired with machine-learning models, including Support Vector Machines, Random Forests, K-Nearest Neighbors, gradient-boosting methods (XGBoost, LightGBM), multilayer perceptrons, and ensemble classifiers, in addition to deep learning architectures, such as convolutional neural networks, LSTMs, BiLSTMs, spatiotemporal graph convolutional networks (ST-GCNs), and transformer-based models (Figure 4).

### 3.4. Analyzed Variables

#### 3.4.1. Gait Variables

Among the 46 included studies, 29 primarily relied on explicit spatiotemporal and kinematic gait variables, such as step length, cadence, stance and swing times, gait cycle parameters, and joint angles.

#### 3.4.2. Raw Coordinates

In contrast, nine studies bypassed handcrafted features altogether and directly analyzed raw joint coordinates or keypoint trajectories using deep learning models, including ST-GCNs, CNN-LSTM hybrids, and transformer-based architectures.

#### 3.4.3. Hybrid Approaches

An additional eight studies adopted hybrid approaches, combining traditional gait metrics with full joint-trajectory inputs to enhance model performance and interpretability.

### 3.5. Evaluation and Validation

**Figure 5:**
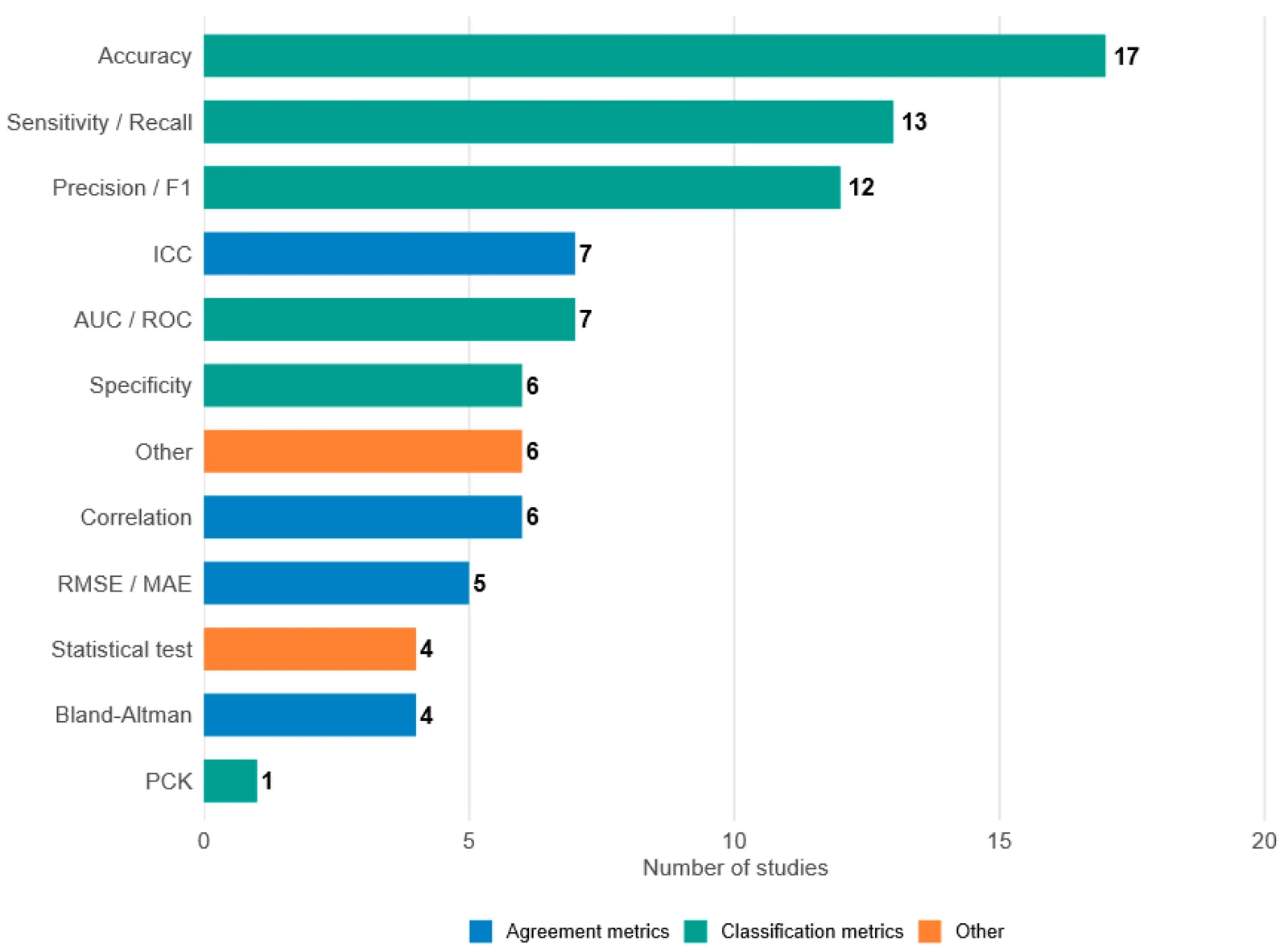
Evaluation approach type distribution and reference systems. Most studies utilized multiple metrics, including intraclass correlation coefficient (ICC), area under the curve/receiver operating characteristic (AUC/ROC), root mean square error/mean absolute error (RSME/MAE), and percentage of correct keypoints (PCK). Some studies are counted in more than one category.

Across the included studies, three distinct evaluation strategies were observed (Figure 5).

First, clinical or label-based validation was the most common approach, taken by 25 studies. These evaluated model outputs against clinical diagnoses or clinician-rated scales, including binary diagnosis labels (e.g., Parkinson’s disease vs. control), disease severity scores (e.g., MDS UPDRS gait), or expert assessments of gait quality. Among these, classification accuracy was the dominant outcome, most often reported as accuracy, sensitivity, specificity, F1 score, or AUC, with sample sizes ranging widely from 14 to more than 400 participants depending on the dataset.

Second, a smaller but technically focused group of 14 lab-based validation studies compared CV-derived gait measures against instrumented or laboratory gold-standard reference systems, such as Vicon motion capture, GAITRite walkways, SMART-DX, IMUs, or pressure-sensing treadmills. These studies typically reported agreement or error metrics, including Pearson or intraclass correlation coefficients (ICC), mean absolute error (MAE), root-mean-square error (RMSE), and Bland–Altman limits of agreement, most commonly for spatiotemporal parameters (step length, stride time, cadence) and joint kinematics (hip, knee, ankle angles).

Finally, a limited subset of six studies did not use an external reference standard. These works relied on internal validation only, such as cross-validation accuracy, repeatability, or reliability analyses (e.g., test–retest ICCs), or proof-of-concept demonstrations without direct comparison to either clinical judgment or biomechanical ground truth.

## 4. Discussion

This scoping review provides a structured overview of how CV has been applied to gait analysis in older adults using conventional video recordings. By synthesizing 46 studies across clinical screening, gait characterization, and validation domains, the findings highlight rapid methodological development and growing interest in low-burden gait assessment tools. However, the evidence mapped in this review does not establish that these tools are deployment-ready or clinically valid. The distribution of study aims highlights both clinical motivations and the emerging development of computer-vision-based gait analysis in older adults (Figure 2).

### 4.1. Current Trends

Screening applications dominated the literature, with a strong emphasis on neurological conditions such as Parkinson’s disease and Alzheimer’s disease, as well as sarcopenia and fall risk (Table 1). This focus on screening aligns with other areas of medicine, including oncology and radiology, that are utilizing electronic health records and patient records to provide specialist-level screening [81]. The frequent use of short walking tasks and timed up-and-go (TUG) videos across these studies reflects a pragmatic alignment with the assessments recommended by the American and British Geriatrics Society and World Falls Guidelines (WFG) Task Force [82]. This alignment suggests that CV could potentially augment familiar assessments, although its clinical benefit and implementation have not yet been established. Subgroup analyses by publication type revealed that conference publications were more likely to prioritize methodological innovation, whereas journal articles more often focused on validation and clinical interpretation (Supplemental Materials).

### 4.2. Scope of Gait Analysis

A substantial subset of studies focused on gait characterization without diagnostic labeling (Figure 3), emphasizing spatiotemporal, kinematic, and variability-based metrics to describe disease-specific gait signatures. These characterization studies frequently overlapped with validation efforts, where computer-vision outputs were compared against gold-standard systems, such as Vicon motion capture or GAITRite walkways, using various validation metrics (Figure 5). These approaches highlight an ongoing methodological trade-off: interpretable, clinically familiar gait metrics versus data-driven representations optimized for predictive performance.

Beyond screening, classification studies expanded this scope to include disease severity grading, mobility level categorization, and differential diagnosis, indicating a growing interest in extracting more granular clinical information from video-based gait data employing recurrent neural networks such as Long Short-Term Memory (LSTM) networks, temporal convolutional networks, graph convolutional networks (GCNs), or more recently, transformer architectures (Figure 4). Pose estimation itself was utilized more as a standardized preprocessing step, allowing researchers to focus on higher-level analysis of gait rather than low-level image processing. This methodology combines mature pose estimation methods with modern learning algorithms.

In addition to studies that focused on the extraction of conventional gait metrics and classification of clinical conditions, a subset of studies utilized raw keypoints, which may be less clinically meaningful given a lack of interpretability. Hybrid models are presented as a stronger application of this framework by combining interpretable gait metrics with data-driven representations; however, their clinical acceptability and utility require prospective validation.

### 4.3. Accessible Methods

Studies predominantly used single commercially available cameras, revealing the aim of accessibility and ease of use when developing and testing new CV applications. The use of commercially available cameras may reduce equipment-related barriers, but scalability in clinical practice has not yet been demonstrated [4]. These methods may also support the development of smartphone-based systems, although their feasibility and effectiveness in resource-limited communities require direct evaluation [83]. The application of CV ranged from open-source, well-established models such as OpenPose to custom-built mobile applications and CV pipelines (Figure 4). While this diversity allows tailored solutions for specific clinical questions, it also complicates cross-study comparison and hampers deployment for clinical translation [84]. Despite frequent claims of real-world feasibility, only six studies evaluated CV systems in uncontrolled home or community environments. Of the 46 studies, we identified only one study [79] that evaluated longitudinal clinical change using repeated video-based gait assessments, and as such, the primarily cross-sectional methods do not demonstrate an impact on patient care due to a lack of construct validity in longitudinal frameworks [85]. Collectively, the evidence more strongly supports technical feasibility and preliminary model performance than immediate clinical applicability.

### 4.4. Limited Validation

The relative scarcity of clinical and lab-based validation-focused studies represents an important gap that must be addressed before video-based gait analysis can be considered for broader clinical use [86]. Greater emphasis on standardized validation protocols, diverse populations, and longitudinal performance will be necessary to determine whether these methods can progress from promising research applications to reliable clinical tools. Some groups published their framework in stages, evaluating the feasibility and then progressing to clinical application [67,69,70,76]. This model accounts for variability in the application and post-processing of the CV method before applying that method to detect and evaluate clinical findings. Furthermore, clinical-label validation does not necessarily establish measurement accuracy. Agreement with diagnostic categories or clinician-rated scales, especially in the absence of lab-based methods, may reflect pathological changes associated with diseases rather than accurate quantification of gait mechanics.

### 4.5. Relevance to Geriatrics

The largest group of excluded studies from this scoping review was due to the wrong patient population, where methods used conventional video recordings but did not focus on older adults (Figure 1). The broad eligibility criteria allowed for inclusion of studies that used large datasets to evaluate age-related changes in gait [55]. Given the unique biomechanics of older adult gait, validation studies specifically with older adults are needed to improve model accuracy [87]. From the included studies, ref. [88] presents a method of fine-tuning a general model for the effects of aging by using a subset of older adult videos. The clinically based studies included in this review were mostly focused on older adults with a specific medical condition (sarcopenia, post-knee arthroplasty, stroke), rather than evaluating healthy older adults in primary care settings. This contributes to concerns from older adults, clinicians, and researchers that AI applications in geriatrics are limited by insufficient validation amongst older adults [86,89].

### 4.6. Limitations

This scoping review may have missed relevant studies due to the databases selected. This review aimed to comprehensively map the methods, outcomes, and validation approaches reported in the literature but did not formally evaluate methodological quality or risk of bias of individual studies, consistent with scoping review methodology [42,47,90]. Therefore, the frequencies reported in this review across clinical conditions should not be interpreted as evidence of effectiveness, accuracy, or clinical superiority. Because age reporting was inconsistent across included studies, studies were screened based on clinical framing and population description rather than a strict chronological age cutoff. Additionally, while the review focused on the use of conventional video recordings, some of the RGB color videos were taken using specialized cameras, which limits the applicability in real-world or clinical settings. Screening bias presented a limitation of this review. The initial title and abstract screening step was performed by a single reviewer due to the volume of records retrieved (n = 1068), which may have contributed to several eligibility decisions that could influence the reliability of the map of evidence presented in this review. Including conference papers and abstracts may have introduced studies with limited methodological detail and preliminary findings that have not undergone rigorous peer review. Additionally, the protocol for the review was not registered, which is a limitation of transparency and may contribute to bias.

### 4.7. Future Work

Future work should prioritize the development of standardized reporting guidelines for CV-based gait studies, including clear documentation of camera setup, participant characteristics, and outcome measures. Greater emphasis on external validation will be necessary to establish measurement credibility, while increased use of real-world and longitudinal study designs can better reflect the contexts in which these tools are intended to operate. Exploration of hybrid modeling approaches that balance interpretability with predictive performance, alongside inclusion of older adults with diverse functional levels and living environments, may further strengthen clinical applicability. If appropriately validated and standardized, CV-based gait analysis using conventional video has the potential to complement and extend existing clinical workflows, especially in settings where access to specialized gait analysis equipment is limited.

## 5. Conclusions

This scoping review examines the use of conventional video recordings for evaluating gait in older adults. It aimed to map the characteristics of this domain using both journal articles and engineering abstracts for a comprehensive review. Through systematic screening, 46 studies were selected. The findings highlighted the distinction between technical feasibility, predictive performance, and clinical validity. The majority of articles utilized low-cost and commercially available cameras to extract spatiotemporal, kinematic, and variability-based metrics and describe disease-specific gait signatures. However, evaluation and validation of these metrics were largely limited to clinical or label-based methods in over half of the included studies, with limited interdisciplinary work utilizing biomechanical gold standards in conjunction with clinical labels. Our findings reveal that conventional video shows promise as an accessible complement to conventional gait assessment, but current evidence does not support its routine clinical use. Standardized reporting and rigorous external, longitudinal, and real-world validation in diverse older adult populations are needed before these tools can reliably inform clinical decision-making.

## Figures and Tables

**Table 1:** Screening studies using standard video recordings in conjunction with short walking tasks. Walking tests are the 5 m walk test (5 MWT), the timed up-and-go test (TUG), and the 10 m walk test (10 MWT).

Title	Screening Condition	Walking Tasks
OpenPose-based Gait Analysis System For Parkinson’s Disease Patients From Arm Swing Data [48]	Parkinson’s	Not reported
Video Based Shuffling Step Detection for Parkinsonian Patients Using 3D Convolution [52]	Parkinson’s	TUG
Model-Based Feature Extraction and Classification for Parkinson Disease Screening Using Gait Analysis: Development and Validation Study [51]	Parkinson’s	TUG
Video-Based Detection of Freezing of Gait in Daily Clinical Practice in Patients With Parkinsonism [54]	Parkinson’s	TUG
Video-based clinical gait analysis in Parkinson’s disease: A Novel approach using frontal plane videos and machine learning [53]	Parkinson’s	10 MWT
A Video-Based Method to Classify Abnormal Gait for Remote Screening of Parkinson’s Disease [49]	Parkinson’s	5 MWT
A two-dimensional video based quantification method and clinical application research of motion disorders [50]	Parkinson’s	5 MWT
SAIL: A deep-learning-based system for automatic gait assessment from TUG videos [55]	Pathological Gait Detection	TUG
Quantitative Gait Feature Assessment on Two-Dimensional Body Axis Projection Planes Converted from Three-Dimensional Coordinates Estimated with a Deep Learning Smartphone App [56]	Pathological Gait Detection	1 m circle
Towards Deep Learning-Based Sarcopenia Screening with Body Joint Composition Analysis [58]	Sarcopenia	Not reported
Non-Invasive Tools for Early Detection and Monitoring of Sarcopenia in Older Individuals [57]	Sarcopenia	TUG
A Simple Fall Detection Scheme for Early Detection of Falls in Elderly People [59]	Fall risk	Not reported
Fall risk prediction using temporal gait features and machine learning approaches [60]	Fall risk	TUG
Mild Cognitive Impairment Detection through Gait Analysis and Standard Cameras [61]	Mild Cognitive Impairment	Curved 10 MWT
Explainable Detection of Alzheimer’s Disease Through Analysis of Human Behavior in Video [62]	Alzheimer’s	TUG

## Data Availability

No new data were created or analyzed in this study. Data sharing is not applicable to this article.

## Supplementary Materials

The following supporting information can be downloaded at https://www.mdpi.com/article/10.3390/healthcare14162487/s1. File S1: Search strings; File S2: Data extraction template; File S3: Summary of each study; File S4: Subgroup analysis by publication type.

## Abbreviations

CV	Computer vision
ADL	Activities of daily living
5MWT	5 m walk test
TUG	Timed up-and-go test
10MWT	10 m walk test
SMPL	Skinned Multi-Person Linear (model)
RGB	Red, green, blue
RGB-D	Red, green, blue–depth
IMU	Inertial Measurement Unit
