# Supplementary material for: Use of Computer Vision with Conventional Video Recordings of Gait in Older Adults: A Scoping Review
Source: Healthcare (Basel). 2026 Aug 11;14(16):2487. doi: 10.3390/healthcare14162487 (PMC13512411; doi:10.3390/healthcare14162487)
Supplement: Supplementary file 1 [file healthcare-14-02487-s001.zip › S1_search_string.pdf]

Database	String
Pubmed	("computer vision"[tiab] OR "Artificial Intelligence"[Mesh] OR "video analysis"[tiab] OR ("deep learning"[tiab] AND "video"[tiab]) OR "pose estimation"[tiab] OR "human motion analysis"[tiab] OR "motion tracking"[tiab] OR "action recognition"[tiab] OR "joint recognition"[tiab] OR "skeleton tracking"[tiab] OR "segmentation"[tiab] OR "semantic segmentation"[tiab] OR "bounding box detection"[tiab] OR "3D pose estimation"[tiab]) AND ("Fall risk"[tiab] OR "fall prevention"[tiab] OR "fall rate"[tiab] OR Mobility[tiab] OR "gait impairments"[tiab] OR "timed-up-and-go test"[tiab] OR "stand-to-sit"[tiab] OR "sit-to-stand"[tiab] OR "gait cycle"[tiab] OR "dynamic balance"[tiab] OR "Gait"[Mesh] OR "Gait Analysis"[Mesh] OR "Accidental Falls"[Mesh]) Filters: from 2020 - 2025
Web of Science	(ALL=(((("Fall risk" OR "fall prevention" OR "fall rate" OR "Accidental Falls") AND ("timed-up-and-go test" OR stand-to-sit OR sit-to-stand OR "dynamic balance" OR mobility)) AND ("computer vision" OR "Artificial Intelligence" OR "video analysis" OR ("deep learning" AND video) OR "pose estimation" OR "human motion analysis" OR "motion tracking" OR "action recognition" OR "joint recognition" OR "skeleton tracking" OR segmentation OR "semantic segmentation" OR "bounding box detection" OR "3D pose estimation")))) AND ALL=(("older adults" OR elderly OR aging OR aged OR senior OR geriatric OR frail OR Aged OR "Frail Elderly"))
IEEE	((("computer vision" OR "Artificial Intelligence" OR "video analysis" OR ("deep learning" AND "video") OR "pose estimation" OR "human motion analysis" OR "motion tracking" OR "action recognition" OR "joint recognition" OR "skeleton tracking" OR "segmentation" OR "semantic segmentation" OR "bounding box detection" OR "3D pose estimation") AND ("Fall risk" OR "fall prevention" OR "fall rate" OR Mobility OR "gait impairments" OR "timed-up-and-go test" OR "stand-to-sit" OR "sit-to-stand" OR "gait cycle" OR "dynamic balance" OR "Gait" OR "Gait Analysis" OR "Accidental Falls")) AND ("older adults" OR elderly OR aging OR aged OR senior OR geriatric OR frail OR "Aged" OR "Frail Elderly"))
Cochrane	("computer vision":ti,ab OR [mh "Artificial Intelligence"] OR "video analysis":ti,ab OR ("deep learning":ti,ab AND video:ti,ab) OR "pose estimation":ti,ab OR "human motion analysis":ti,ab OR "motion tracking":ti,ab OR "action recognition":ti,ab OR "joint recognition":ti,ab OR "skeleton tracking":ti,ab OR segmentation:ti,ab OR "semantic segmentation":ti,ab OR "bounding box detection":ti,ab OR "3D pose estimation":ti,ab) AND ("Fall risk":ti,ab OR "fall prevention":ti,ab OR "fall rate":ti,ab OR Mobility:ti,ab OR "gait impairments":ti,ab OR "timed-up-and-go test":ti,ab OR stand-to-sit:ti,ab OR sit-to-stand:ti,ab OR "gait cycle":ti,ab OR "dynamic balance":ti,ab OR [mh Gait] OR [mh "Gait Analysis"] OR [mh "Accidental Falls"])
