# Supplementary material for: Use of Computer Vision with Conventional Video Recordings of Gait in Older Adults: A Scoping Review
Source: Healthcare (Basel). 2026 Aug 11;14(16):2487. doi: 10.3390/healthcare14162487 (PMC13512411; doi:10.3390/healthcare14162487)
Supplement: Supplementary file 1 [file healthcare-14-02487-s001.zip › S2_Extraction_Template.pdf]

General information

Title

Title of paper / abstract / report that data are extracted from

Country in which the study conducted

1. United States
2. UK
3. Canada
4. Australia
5. China
6. Other

Notes

Characteristics of included studies

Methods

Aim of study

Study design

1. Randomised controlled trial
2. Non-randomised experimental study
3. Cohort study
4. Cross sectional study
5. Case control study
6. Systematic review
7. Qualitative research
8. Prevalence study
9. Case series
10. Case report
11. Diagnostic test accuracy study
12. Clinical prediction rule
13. Economic evaluation
14. Text and opinion
15. Other

Type of paper

1. Conference paper
2. Abstract
3. Journal article
4. Preprint
5. Other

Participants

Inclusion criteria

Exclusion criteria

Total number of participants

Mean age

Setting

1. Home
2. Lab
3. Clinical setting (hospital, therapy)
4. Outdoors
5. Other

Participant types

1. Healthy community-dwelling older adults
2. Parkinson's disease
3. Alzheimer's disease
4. Osteoarthritis
5. Nursing home resident
6. Cognitive impairment
7. Other

Method of recruitment of participants

1. Phone
2. Mail
3. Clinic patients
4. Voluntary
5. Other

Video Aquisition

Type of video recording

1. Video recorder (GoPro, Sony, etc)
2. Specialized camera only using RGB data (Kinect, etc)
3. Smartphone camera
4. Smartphone application
5. Other

Number of cameras

Recording Metrics

	Angle	Frame rate	Resolution
**Metric**			

Computer Vision Methodology

Pose estimation method

Key point information (number of joints)

Dimension

1. 2D
2. 3D

Preprocessing methods

Reported gait metrics

	Metrics
**Extracted joints/joint angles**	
**Spatiotemporal**	
**Kinematic**	
**Stability**	
**Variability**	
**Other derived metrics**	

Modeling/Validation

Machine learning models used (CNN, LSTM, etc.)

1. SVM
2. RF
3. CNN
4. LSTM
5. Transformer
6. Other

Feature selection methods

Target Metric

Evaluation Metric

Methods of validation (insoles, force plate, etc)

Clinical relevance

Clinical assessment of gait (TUG, 6-meter walking test, etc)

Clinical variables of interest (frailty, Parkinson's severity, fall risk, dynamic balance, etc)
