# Supplementary material for: Use of Computer Vision with Conventional Video Recordings of Gait in Older Adults: A Scoping Review
Source: Healthcare (Basel). 2026 Aug 11;14(16):2487. doi: 10.3390/healthcare14162487 (PMC13512411; doi:10.3390/healthcare14162487)
Supplement: Supplementary file 1 [file healthcare-14-02487-s001.zip › S4_Subgroup_Analysis.pdf]

Study Characteristics	Age	Sample size	Setting
Journal Articles (n=30)	69.4 ± 5.5	84.24 +/- 112.63	13 (lab), 8 (clinical), 2 (residential care), 8 (other/not specified)
Conference Papers (n=16)	63.8 ± 6.6	84.8 +/- 109.6	6 (lab), 7 (clinical), 2 (residential care), 1 (gymnasium)

Study Aims	Journal Articles (n=30)	Conference Papers (n=16)
Screening	8	7
Classification	8	6
Gait Characterization	14	2
Person Identification	0	1

Type of Video Recording	Journal Articles (n=30)	Conference Papers (n=16)
Smartphone camera	10	4
Video recorder (GoPro, Sony, etc.)	13	8
Specialized RGB camera system*	4	3
CCTV / surveillance camera	2	0
Not specified	1	1

Feature Representation	Journal Articles (n=30)	Conference Papers (n=16)
Explicit gait variables	21	8
Raw keypoints/joint trajectories	4	5
Hybrid approaches	5	3

Validation Type	Journal Articles (n=30)	Conference Papers (n=16)
Clinical validation	14	11
Lab-based validation	11	3
No validation	5	1
Person-identification evaluation	0	1
